# Epilepsy and psychosis: navigating through a complex intersection

**DOI:** 10.1192/bjo.2025.70

**Published:** 2025-06-26

**Authors:** Marco Mula, Andres M. Kanner, Allan H. Young, Annabella Di Giorgio, Andreas Schulze-Bonhage, Eugen Trinka

**Affiliations:** Department of Neurology, St George’s University Hospital, London, UK; City St George’s, University of London, London, UK; Epilepsy Division and Comprehensive Epilepsy Center, Department of Neurology, University of Miami, Miller School of Medicine, Miami, Florida, USA; Department of Psychological Medicine, Institute of Psychiatry, Psychology and Neuroscience, King’s College London, London, UK; South London and Maudsley NHS Foundation Trust, Bethlem Royal Hospital, London, UK; Department of Mental Health and Addictions, ASST Papa Giovanni XXIII, Bergamo, Italy; Epilepsy Center, University Medical Center, University of Freiburg, Member of European Reference Network EpiCARE, Freiburg, Germany; Department of Neurology, Neurocritical Care and Neurorehabilitation, Member of European Reference Network EpiCARE, Centre for Cognitive Neuroscience, Christian Doppler University Hospital, Paracelsus Medical University, Salzburg, Austria; Neuroscience Institute, Centre for Cognitive Neuroscience, Christian Doppler University Hospital, Paracelsus Medical University, Salzburg, Austria; Karl Landsteiner Institute of Neurorehabilitation and Space Neurology, Salzburg, Austria

**Keywords:** Epilepsy, psychosis, antiseizure drugs, antipsychotic drugs, lurasidone

## Abstract

**Background:**

The prevalence of psychiatric disorders in people with epilepsy is as high as 43% and, among them, psychoses represent a severe comorbidity.

**Aims:**

This is a narrative review discussing the interplay between epilepsy and psychosis and identifying challenges in diagnosing and managing psychotic symptoms in epilepsy, focusing on the past 10 years.

**Method:**

Articles published between June 2014 and December 2024 were identified through searches in PubMed using the search terms ‘psychosis’, ’seizure, epilepsy and convulsion’, ‘epile*’, ’seizure*’ and ‘convuls*’.

**Results:**

The association between epilepsy and psychosis was shown to be bidirectional, with people with psychosis being at increased risk of epilepsy. In epilepsy, psychotic symptoms may occur in three clinical scenarios, with clinical presentation and management varying in relationship to these: seizure-related (peri-ictal), treatment-related or independent of the former.

**Conclusions:**

There are no guidelines for the management of psychotic symptoms in epilepsy, but it is possible to apply policies for the treatment of psychoses, taking into account the peculiarities and needs of people with epilepsy.

The first known document reporting the association between epilepsy and psychosis dates back to the second millennium BC, with an Assyrian medical text (Assyrian Medical Texts, AMT 96,7 British Museum and Keilschrifttexte aus Assur Religiösen Inhalts KAR 26, Berlin).^1,2^ Subsequently the Greeks (e.g. Hippocrates) described the association between epilepsy and psychiatric disorders as arising in the brain and influenced by the moon.^3^ In 20th-century neuropsychiatry, many authors have shown interest in the psychoses of epilepsy, including Slater, Landolt and Trimble, who introduced new concepts such as interictal schizophrenia-like psychoses of epilepsy, *Forcierte Normalisierung* and *Ersatzpsychose* (forced normalisation and alternative psychoses), providing accurate descriptions of clinical presentations and describing long-term outcomes.^3–8^

With the new definition of epilepsy, psychiatric disorders become an integral part of the condition itself, leading to a rejuvenated interest in psychiatric disorders.^9^ Furthermore, the new multi-axial classification of epilepsy syndromes recognises the importance of comorbidities, along with aetiologies and seizure types.^10^ However, data on the management of psychotic symptoms in epilepsy remain limited, and clinical management is mainly based on individual experience or uncontrolled studies.

This is a narrative review discussing the interplay between epilepsy and psychosis and identifying challenges in diagnosing and managing psychotic symptoms in adults with epilepsy. Articles published between June 2014 and December 2024 were identified through searches in PubMed using the search terms ‘psychosis’, ’seizure, epilepsy and convulsion’, ‘epile*’, ’seizure*’ and ‘convuls*’. No language restrictions were applied. This search generated 749 abstracts. Articles were selected based on originality and relevance to the present topic. Additional articles were identified from the authors’ files and chosen bibliographies.

## Epidemiology

The prevalence of any psychiatric disorder in unselected samples of adults with epilepsy is reported to be as high as 43.3%. The most frequent examples include mood disorders (up to 40% for lifetime diagnoses and up to 23% for current) and anxiety disorders (30.8% for lifetime and up to 15.6% for current). Psychotic disorders are also over-represented in epilepsy, in the region of 4%.^11,12^ Psychiatric disorders have previously been shown to have a high prevalence in individuals newly diagnosed with epilepsy,^13^ and a complex relationship with somatic’ disorders has been hypothesised.^14^

Temporal lobe epilepsy, in particular, has long been recognised as a risk factor for psychosis. However, there is a lack of consistency in findings across studies on the effect size of this risk, which reflects methodological differences in studies and changes in diagnostic classifications within the disciplines of neurology and psychiatry. Various studies have adopted different definitions and classifications for psychosis. Within these limitations, a meta-analysis of 58 studies reported psychotic disorders (from schizophrenia to schizophreniform disorders) in up to 6% of people with epilepsy, which represents an almost threefold increased risk as compared with the general population, rising to eightfold in patients with temporal lobe epilepsy.^15^ Two recent meta-analyses provided similar estimates confirming previous observations.^16,17^ Nevertheless, patients with epilepsy present with an increased (two- to threefold) risk of being hospitalised for schizophrenia as compared with the general population.^18^ Taken together, all of the above data clearly support the premise that epilepsy, involving the temporal lobe type in particular, represents a risk factor for the development of psychosis.

However, although the relationship between epilepsy and psychoses has been shown not to be simply unidirectional, people with psychoses have also been shown to be at increased risk of developing epilepsy. A matched, longitudinal cohort study including 3773 people with epilepsy and 14,025 controls, matched by year of birth, sex, general practice and years of medical records prior to the index date, indicated an incidence rate ratio (IRR) of psychosis, depression and anxiety significantly increased for all years preceding epilepsy diagnosis (IRR, 1.5–15.7) and following (IRR, 2.2–10.9), suggesting a bidirectional relationship between epilepsy and psychiatric disorders, including psychoses.^19^

A Swedish population-based case-control study involving 1885 subjects with new onset of unprovoked seizures and 15 080 random controls showed an age-adjusted odds ratio for unprovoked seizures of 2.3 following a hospital discharge for psychosis for >2 years.^20^ A retrospective cohort study investigating the co-occurrence of epilepsy and psychosis showed a two- to threefold increased risk of epilepsy in people admitted to hospital with a diagnosis of schizophrenia, and a four- to fivefold increased risk of schizophrenia in those admitted with a diagnosis of epilepsy.^21^ Taking these data together, it is now recognised that epilepsy is between four and six times more frequent among individuals with psychosis and thought disorders as compared with the general population.^18^

However, further research in this area is needed. No gender or age difference was identified across studies, but there remains a lack of data about the role of demographic variables, particularly ethnicity and special populations. Furthermore, numbers of studies looking at multiple psychiatric comorbidities in epilepsy remain minimal. One recent meta-analysis showed that depression and anxiety comorbidity is more frequent in people with epilepsy than without (odds ratio 3.7, 95% CI 2.1–6.5, *P* < 0.001, *I* ^2^ = 0%, Cochran Q *P*-value for heterogeneity 0.84), as well as the association between depression and attention-deficit hyperactivity disorder (odds ratio 5.2, 95% CI 1.8–15.0, *P* = 0.002, *I* ^2^ = 0%, Cochran Q *P*-value for heterogeneity 0.79), but data on psychosis were not available.^22^ However, current data do not suggest, for example, that depression in epilepsy is more likely to be associated with psychotic symptoms than in the general population.^23,24^

## Pathophysiology

The neurobiological links between epilepsy and psychoses have not been clarified. First, it is essential to point out that the relationship between epilepsy and psychosis as brain disorders is different from that between psychotic symptoms and seizures, and the epidemiological data supporting the bidirectional relationship looked specifically at the two brain conditions. This bidirectional relationship between these two disorders suggests shared or common neurobiological mechanisms, and data on temporal lobe epilepsy seem to indicate that psychoses may represent a marker of severity of temporal lobe pathology, given the number of abnormalities identified in the mesiotemporal structures.^25–28^ However, a systematic review of the structural and functional neuroimaging studies on the psychosis of epilepsy shows that brain abnormalities in individuals with epilepsy and psychoses go beyond the mesiotemporal structures, involving networks that may be distant, but still connected, to the frontotemporal networks.^29^ In terms of epilepsy, an early onset of epilepsy and a long duration of active disease are also well-recognised epilepsy-related risk factors.^30^ Data on the relative contribution of additional neurological factors, such as dementia or stroke, for example, are lacking despite their potential role, given that they are also associated with an increased risk of epilepsy.^31–33^

In the same way, there are no data about risk factors commonly identified for psychosis outside epilepsy, such as alcohol and drug use and family history, but it is reasonable to hypothesise that these can represent contributory factors. Finally, the emerging role of neuroinflammation in both epilepsy and psychosis has to be acknowledged. Neuroinflammation can act as a key contributor to the development and progression of these conditions, by creating an environment of excessive neuronal excitability through the activation of glial cells, which can lead to seizures in epilepsy and contribute to the distorted perceptions and thought patterns seen in psychosis; essentially, chronic inflammation in the brain can disrupt regular neural communication and function, leading to symptoms of these disorders.^34^ There is no doubt that psychosis in epilepsy remains understudied, and studies are biased by small sample sizes and antipsychotic use^35^ (Table 1).

**Table 1:** Risk factors for the development of psychosis in epilepsy

Epilepsy-related	Patient-related	Others
Temporal lobe pathology (e.g. hippocampal sclerosis, low-grade tumours) Early onset Long duration Active epilepsy Drug-resistant	Family history of psychosis Alcohol abuse Drug use/abuse	Other structural brain lesions (e.g. stroke) Other neurodegenerative conditions (e.g. dementia)

At this stage, it is essential to point out that psychotic symptoms and seizures have historically shown an antagonistic relationship, meaning that the neurobiology of seizures seems to antagonise that of psychotic symptoms. One of the first observations dates back to the seminal work of Ladislas Meduna,^36–41^ representing the theoretical basis for the subsequent development of shock therapies.^43^ In 1934, Meduna induced an epileptic seizure in a 33-year-old man with catatonic schizophrenia. Following 5 further seizures over 3 weeks, the psychosis was relieved. Meduna also observed greater concentrations of brain glia in individuals with epilepsy than in those with schizophrenia and, in his monograph Die Konvulsionstherapie der Schizophrenie, reported that more than half of 110 schizophrenic individuals recovered following seizures induced by pentylenetetrazol.^41^ By 1936, pentylenetetrazol-induced seizures were in use throughout the world, with electrical inductions later replacing pharmacologically induced seizures, opening the field for electroconvulsive therapy.

## Clinical presentation

In people with epilepsy, psychotic symptoms may occur in three major clinical scenarios: seizure-related (peri-ictal), interictal and treatment-related (Table 2).

**Table 2:** Psychotic symptoms in epilepsy

Seizure-related (peri-ictal)	Seizure-unrelated (interictal)	Treatment-related
Pre-ictal Ictal Post-ictal	Schizophrenia and related disorders Schizophrenia-like psychosis of epilepsy Other clinical syndromes (e.g. autoimmune encephalitis)	Toxicity or adverse event of antiseizure medications Forced normalisation (antiseizure medicines, neurostimulation, epilepsy surgery)

### Peri-ictal psychoses

Among peri-ictal psychoses, pre-ictal ones are the least common and least studied. These present with various unspecific symptoms during the hours (rarely up to 3 days) before a seizure, including derealisation and depersonalisation experiences, forced thinking, ideomotor aura, déjà vu, jamais vu, anxiety, euphoria and perceptual experiences such as hallucinations or illusions.^44^ These symptoms usually end with the seizure but are not associated with detectable electroencephalogram changes.^45^

Ictal psychoses are episodes of non-convulsive status epilepticus, mostly of temporal lobe origin, but they represent an infrequent occurrence.^46,47^

### Post-ictal psychoses

Among all post-ictal symptoms, psychotic ones occur in only 4% of cases while headaches and migraines are much more frequent.^48^ However, when looking at peri-ictal psychotic symptoms, post-ictal ones are the most frequent, representing 60% of peri-ictal psychotic symptoms.^45,49,50^ The classic clinical presentation is characterised by a period of normal mental state, known as the lucid interval, following a seizure or cluster of seizures and lasting between 24 and 48 h.^51–53^ The psychotic episode is characterised by mixed moods with psychomotor excitation, ecstatic moods and mystic or religious delusions,^26^ resembling more an affective psychosis than a pure psychotic disorder.^54^ However, post-ictal psychoses differ from depressive disorders with psychotic symptoms. While both post-ictal psychosis and psychotic symptoms in depression involve experiencing psychosis, the former occurs directly following a seizure, the psychotic symptoms do not necessarily follow the mood theme and, while post-ictal psychosis is usually temporary, resolving within days or weeks, psychotic depression may last longer depending on treatment.

Post-ictal psychoses represent psychiatric emergencies, given the high prevalence of aggressivity and mortality rates for suicide or other accidents historically reported in up to 28.5% of cases.^55^ Recent data on the prognosis of post-ictal psychoses are still lacking. In terms of long-term prognosis, historical data suggest the development of chronically unremitting psychotic symptoms in 21–25% of individuals.^5,55^ More recent data indicate the possibility of bimodal psychosis characterised by relapses, against a background of chronic symptoms.^18^

### Interictal psychoses

Historical descriptions of chronic psychosis in epilepsy include the so-called interictal schizophrenia-like psychosis of epilepsy, which various authors have identified as distinct from sporadic schizophrenia in terms of clinical presentation and long-term outcomes.^56,57^ In fact, according to historical descriptions, psychoses in epilepsy seem to be characterised by low rates of unfavourable symptoms, low rates of cognitive impairment, better response to treatment and lower rates of hospitalisation.^49,57^ Risk factors for the development of chronic psychosis in epilepsy include temporal lobe epilepsy, at least 15 years’ duration of active disease and a family history of psychosis.^30^ Adachi et al have pointed out that the course of the disease differs between patients with interictal psychoses as compared with those with schizophrenia, with a progressive deterioration occurring only in the latter.^58^

### Treatment-related psychoses

A retrospective study involving 2630 people with epilepsy between 1993 and 2015 looked specifically at the aetiology of psychotic symptoms; only one out of seven cases showed a clear temporal relationship with the treatment. All individuals showed complete remission following drug discontinuation, with risk factors including a diagnosis of temporal lobe epilepsy, female gender and treatment with levetiracetam.^59^ One US retrospective study of 4085 adults newly started on antiseizure medication (ASM) found that around 17.2% developed psychiatric side-effects with any drug, with risk factors including a diagnosis of drug-resistant epilepsy and a previous psychiatric history.^60^ Psychotic symptoms have been described with most ASMs; sodium channel blockers are those with the lowest risk of psychiatric side-effects.^61^ Cenobamate has a dual mechanism of action, blocking voltage-gated sodium channels through a pronounced action on persistent rather than transient currents and acting as a positive allosteric modulator of GABA_A_ receptors independently from the benzodiazepine binding site.^62–64^ Cenobamate has shown a low prevalence of psychiatric side-effects and a good overall tolerability profile.^65^

With regard to ASM-related psychotic symptoms, forced normalisation plays a unique role. This is an intriguing phenomenon characterised by the emergence of psychiatric disturbances following either the establishment of seizure control or reduction in epileptic activity in an individual with previous uncontrolled epilepsy.^66^ The mechanism is still unknown, but has been described with both ASM and vagus nerve stimulation, raising the hypothesis that the phenomenon is linked to the neurobiology of seizure control.^67,68^ Finally, one recent systematic review clarified that forced normalisation can be seen primarily in focal epilepsies (80%) and symptomatic aetiology (44%).^66^ Studies looking at the neurobiological mechanisms of forced normalisation would be able to elucidate the neurobiological links between epilepsy and psychosis.

Postoperative de novo psychoses represent specific conditions with hitherto minimal data; epidemiological data remain sparse, but these represent a serious complication of epilepsy surgery.^69–71^

### Diagnosing psychotic disorders in epilepsy

Given the complexities of clinical presentations, it can be challenging for neurologists to identify early psychotic problems, especially for those with limited psychiatric training (Table 3).

**Table 3:** Clinical elements requiring consideration when diagnosing interictal psychosis in epilepsy

Clinical history	Diagnosis of temporal lobe epilepsy Family history of psychosis 10 years’ latency between onset of the epilepsy and that of psychosis Multiple episodes of post-ictal psychosis
Clinical symptoms	Unchanged or stable personality Material specific deficits (e.g. declarative memory) Persecutory or religious themes Non-flat affect Mood symptoms
Response to treatment	Good response to antipsychotic drugs Good long-term functioning

The prompt identification of symptoms represents the foremost step for a prompt referral to psychiatric services. There is only one screening instrument proposed for the identification of psychotic symptoms in epilepsy, namely the Emotions with Persecutory Delusions Scale (EPDS).^72^ This self-reported questionnaire has shown good psychometric properties against clinical diagnosis. Nevertheless, the lack of a clear cut-off score and the complex scoring system significantly affect implementation in clinical practice. At present, there are no recommended screening instruments for psychotic symptoms in epilepsy. Clinicians, invividuals and their families need to be aware of the increased risk and potential clinical scenario, and neurologists should consider exploring with simple questions the presence of hallucinations or thought disorders for prompt referral of patients to mental health services. Patients with intellectual disabilities and epilepsy represent a unique population posing several challenges in terms of diagnosis and management of psychotic symptoms and, to date, data are still lacking.^73^ From a psychiatric perspective, it can be challenging for psychiatrists not trained in the neuropsychiatry of epilepsy to immediately distinguish epilepsy-related presentations (e.g. post-ictal psychosis or forced normalisation) from comorbid schizophrenia or schizophreniform disorders, and to distinguish between episodic versus chronic conditions. Understanding the nature of these conditions is vital to inform optimal treatments. The field of neuropsychiatry, as a subspecialty, clearly represents the opportunity to provide a comprehensive approach to clinical problems such as this, bridging between neurology and psychiatry.^74^

## Treatment issues

No evidence-based clinical practice guidelines exist for the management of psychotic symptoms in epilepsy, and evidence on the effectiveness of antipsychotic drugs in the treatment of psychotic symptoms in epilepsy is inconclusive.^75^ During the past 10 years, several expert opinion papers have attempted to guide the management of these symptoms.^76,77^ In general terms, it is reasonable to apply guidelines of treatment for psychoses outside epilepsy – keeping in mind the specific needs of this unique population of individuals, namely pharmacological interactions of psychotropic drugs with ASMs and the risk of seizure worsening with antipsychotic medications.

### Interactions and seizure risk

Regarding kinetic interactions, ASMs with enzyme induction properties such as phenytoin, carbamazepine and barbiturates reduce the blood levels of almost all antipsychotic drugs.^61^ However, this interaction is particularly relevant for quetiapine because it is metabolised only by the enzyme CYP3A4, and concomitant use with any inducer would lead to almost undetectable blood levels below 700 mg.^78^ Individual differences in treatment response should be carefully considered in regard to olanzapine and clozapine, which have complex metabolisms with multiple enzymatic pathways involved. Conversely, not all antipsychotics appear to influence the enzymatic pathways of ASMs significantly and, consequently, do not appear to affect ASM blood levels.^45^

Data on pharmacodynamic interactions are generally limited, but it is essential to consider the implications of combining antipsychotics and ASMs with a similar spectrum of side-effects, especially for sedation, weight gain and cardiotoxicity; and the apparent risk of bone marrow suppression in regard to the combination of carbamazepine and clozapine.^45^

Antipsychotic drugs have always been considered as being associated with an increased risk of seizure worsening. In psychiatric practice, antipsychotic medications are mainly chosen for their side-effect profile, in particular the propensity for extrapyramidal side-effects, weight gain or arrhythmias,^79^ but it is obvious that the issue of seizures is particularly evident in regard to people with epilepsy.

A systematic review of Food and Drug Administration (FDA)-controlled trials of antipsychotic drugs showed that clozapine is associated with the highest risk of seizures, with a standardised incident ratio of 9.5 (7.27–12.20) as compared with placebo, and such a risk seems to be dose and titration dependent.^80^ Olanzapine and quetiapine were also considered to carry some risk, with an incident ratio in the region of 2.50 (1.58–3.74) for olanzapine and 2.05 (1.21–3.23) for quetiapine.^80^ However, a very recent network meta-analysis showed that all second-generation antipsychotics, including even clozapine, are associated with no significantly increased risk of seizure occurrence as compared with placebo.^81^ This discrepancy with previous studies may be related to the adoption of high dosages and rapid titrations, which are not typically current standard of care. It is important to emphasise that all the above data are derived from psychiatric samples rather than from individuals with epilepsy. It is, therefore, evident that the issue of seizures as a side-effect of antipsychotics still deserves further clarification, and careful clinical monitoring is crucial in regard to people with epilepsy and psychosis. Among recent antipsychotics, lurasidone is a dopamine type 2 (D2), serotonin type 2 (5-HT2A) and 5-HT7 receptor antagonist^82^ and showed a favourable side-effect profile with a very low rate of side compared with placebo.^83^

### Treatment duration

Regarding psychosis in epilepsy, treatment requirements and duration represent a challenge because the time course of psychosis in epilepsy differs from that of new-onset psychosis and depends on the type of psychosis (Table 2). Post-ictal psychotic episodes in epilepsy are more likely to be recurrent, while interictal psychoses are more likely to be chronic.

Regarding post-ictal psychosis, an international Delphi survey among a group of experts suggested that symptomatic antipsychotic treatment is generally warranted but should subsequently be tapered off (e.g. after some weeks), given the episodic nature of the condition.^76^ Moreover, given the condition’s self-limiting time course, it must be acknowledged that, in many cases, minor episodes can also be managed by observation, nursing or carer supervision.^84^ In many instances there is no need for long-term antipsychotic treatment, which is mainly prescribed to reduce mortality and morbidity;^45^ however, in one out of four cases, post-ictal psychosis may progress to a chronic psychosis,^85^ and this would pose the question of long-term treatment with antipsychotics. Complete seizure control would represent the best preventive treatment for post-ictal psychoses and the long-term development of chronic psychoses. However, there are isolated case reports of individuals who developed psychosis even years following successful epilepsy surgery.^85^

For other peri-ictal psychoses, psychotropic medications are not generally indicated^45^ and resolve with effective management of the epilepsy, with no need to treat the psychosis directly.

Finally, the treatment of chronic interictal psychosis tends to be similar to that of primary schizophrenia, i.e. long term, with preferential use of antipsychotic medications, paying attention to the risk for interactions and seizure relapse.^76,77^ To date, no studies have specifically investigated depot or long-acting injectable antipsychotics in people with epilepsy, or whether these are associated with an increased risk of seizure deterioration as compared with oral formulations.

In conclusion, psychosis in epilepsy still represents a clinically relevant comorbidity deserving clinical attention. The relationship between these two conditions is complex and multifaceted, involving both shared neurobiological mechanisms and the impact of seizures on mental health. Epilepsy, in particular temporal lobe epilepsy, has been linked to an increased risk of psychotic disorders, but such a relationship is bidirectional. The precise mechanisms involved remain incompletely understood, but multiple factors are likely to be in play, from the involvement of specific brain structures or networks to neurochemical imbalances and individual genetic predisposition, all contributing to this co-occurrence. Treatment approaches often involve antipsychotic medications, although managing both conditions simultaneously can be challenging due to potential drug interactions and side-effects. Early identification and a holistic, individualised treatment plan are crucial for improving outcomes and quality of life for individuals affected by both epilepsy and psychosis.

## Data Availability

Data availability is not applicable to this article as no new data were created or analysed in this study.
